# Hormone replacement therapy and the risk of achalasia in postmenopausal women: A nationwide cohort study

**DOI:** 10.1097/MD.0000000000047834

**Published:** 2026-02-28

**Authors:** Mi Jin Oh, Yoon Jin Choi, Kyungdo Han, Kyu-Na Lee, Gi Hong Park, Bokyung Kim, Hyunsoo Chung, Sang Gyun Kim, Soo-Jeong Cho

**Affiliations:** a Department of Internal Medicine and Liver Research Institute, Seoul National University College of Medicine, Seoul, Republic of Korea; b Department of Internal Medicine, National Cancer Center, Goyang, Republic of Korea; c Department of Statistics and Actuarial Science, Soongsil University, Seoul, Republic of Korea; d Department of Public health, Catholic University, Seoul, Republic of Korea; e Department of Internal Medicine, Seoul Metropolitan Government Seoul National University Boramae Medical Center, Seoul, Republic of Korea.

**Keywords:** achalasia, hormone replacement therapy, risk factor

## Abstract

Achalasia is an esophageal motility disorder characterized by progressive dysphagia, leading to a significant decline in the quality of life of affected individuals. This study aimed to investigate hormone replacement therapy (HRT) as a risk factor for achalasia in postmenopausal women. Postmenopausal women who underwent healthcare screening in 2009 through the Korean National Health Insurance Service were enrolled and followed up till December 31, 2022. The risk of achalasia in relation to history and duration of HRT (none, <2 years, 2–5 years, and over 5 years) was calculated using adjusted hazard ratios with 95% confidence intervals. Subgroup analyses were conducted based on age, income, history of autoimmune diseases, and female reproductive factors. The overall cumulative incidence of achalasia in the study cohort was 731 of 1,101,080 subjects. Across all adjusted models, the risk of developing achalasia was significantly higher in participants who received HRT than in those with no history of HRT. The risk increased in a dose-dependent manner with the HRT duration, reaching an adjusted hazard ratio of 1.47 (95% confidence interval: 1.25–1.73) in those with prolonged use of HRT for 5 years or longer. Such a trend remained consistent in the subgroup analyses. In this study, HRT use was associated with an increased risk of achalasia in a dose-dependent manner. These findings underscore the need for vigilant monitoring of esophageal symptoms in postmenopausal women with a prolonged history of HRT.

## 1. Introduction

Achalasia is a motility disorder of the esophagus characterized by impaired relaxation of the lower esophageal sphincter and the absence or spastic peristalsis of the esophageal body.^[1–3]^ Common symptoms include progressive dysphagia of both liquids and solids, chest pain, regurgitation, coughing, painful swallowing, and epigastric discomfort. When left untreated, patients may experience malnutrition and weight loss due to these eating-related issues.^[4,5]^ After the introduction of high-resolution manometry, there has been a significant improvement in the diagnostic accuracy of achalasia, resulting in a global rise in its incidence.^[6,7]^ With an incidence of 1 to 5 cases per 100,000 person-years,^[5]^ the chronic nature of achalasia and its substantial impact on eating abilities contribute to poor quality of life and impaired functional capacity in patients.^[8]^

The development of achalasia is associated with the degeneration of ganglion cells in the lower esophagus due to inflammatory responses, as evidenced by studies on esophageal biopsies or surgical samples from patients with achalasia.^[9,10]^ Although the exact cause remains unclear, previous research has suggested that this degeneration of ganglion cells may occur through an autoimmune process initiated by latent viral infections in genetically predisposed individuals.^[5,9,11,12]^ Furthermore, small case–control studies have indicated associations between autoimmune conditions, viral infections, and low socioeconomic status with an increased risk of achalasia.^[13,14]^ Despite these findings, the pathophysiology of achalasia remains poorly understood, and no modifiable risk factors have been identified to date.

Female sex hormones have a multifaceted, yet significant, association with autoimmune disorders.^[15,16]^ Most rheumatic diseases exhibit a female preponderance, and factors such as pregnancy, breastfeeding, oral contraceptive (OC) use, and hormone replacement therapy (HRT) can influence the development and prognosis of these conditions.^[15,16]^ Considering that autoimmune reactions are considered the key mechanisms in the pathophysiology of achalasia, we hypothesized that female sex hormones might play a role in its development. To explore this further, we investigated the effect of HRT on postmenopausal women using data from the Korean National Health Insurance Service (NHIS).

## 2. Methods

### 2.1. Data source and study population

We analyzed the anonymized data provided by the Korean National Health Insurance Sharing Service. The NHIS is a national insurance service in Korea that covers approximately 97% of the whole population. After incorporating the healthcare claims data of the Medical Aid program from 2006, the NHIS data represent the entire South Korean population. The NHIS provides biennial general health screening and national cancer screening for its enrollees, enabling the collection of laboratory results, anthropometric measurements, self-reported data on health-related behaviors, female reproductive factors, and histories of HRT use.

**Figure 1.**
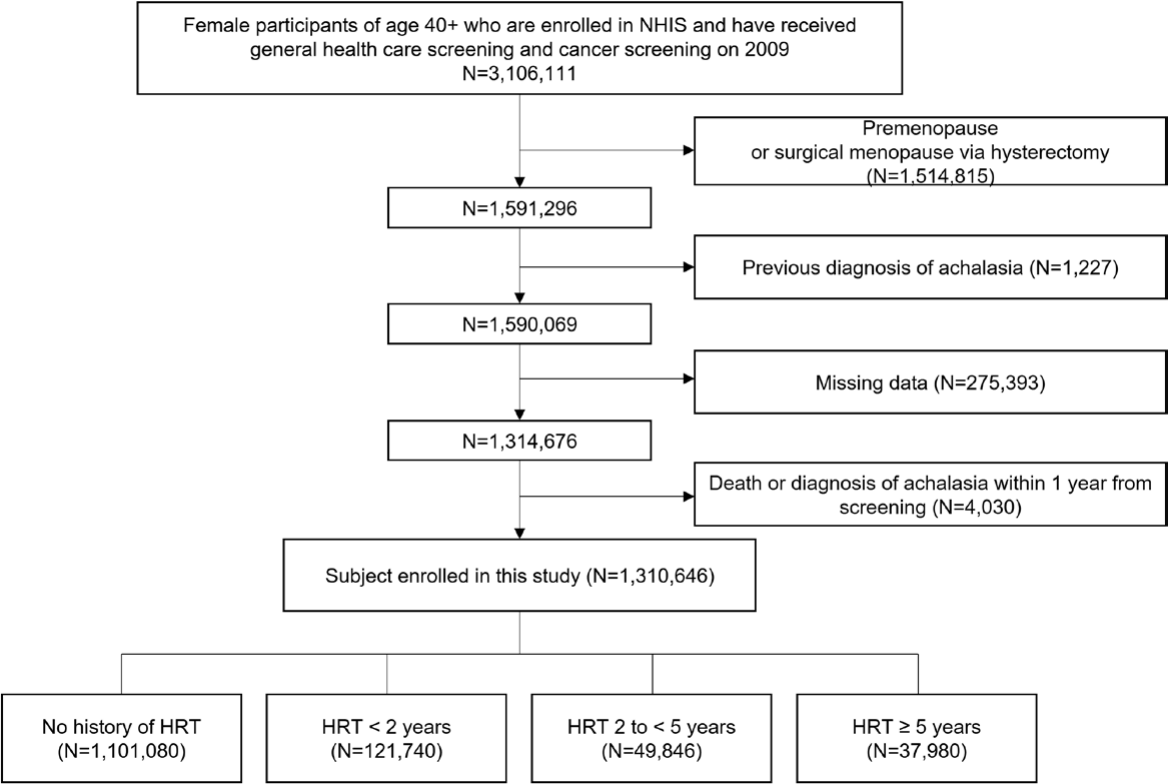
Study flow showing the enrollment process. HRT = hormone replacement therapy, NHIS = National Health Insurance Service.

Women aged 40 years or older who underwent general healthcare screening and cancer screening in 2009 were included in this study (Fig. 1). Women who were premenopausal or had undergone menopause after hysterectomy (n = 1,514,815) and those with a previous diagnosis of achalasia (n = 1227) were also excluded. Enrollees with missing data on female reproductive factors (n = 207,247) and other study variables (n = 68,146) were excluded. A comparative analysis of these excluded individuals (n = 275,393) is presented in Table S1, Supplemental Digital Content, https://links.lww.com/MD/R454, to assess the potential selection bias. Finally, to apply a 1-year lag period, individuals who were diagnosed with achalasia or died within 1 year from the date of general healthcare screening were excluded (n = 4030). Ultimately, a cohort of 1,310,646 postmenopausal women were included in the study and divided into 4 groups according to their self-reported history of HRT: no history of HRT, history of HRT for <2 years, HRT for between 2 and 5 years, and HRT for 5 years or longer. The cohort was followed up until the diagnosis of achalasia, death, or the end of the study period (December 31, 2022), with a mean follow-up duration of 11.89 ± 1.98 years.

This study was exempted from review by the Institutional Review Board (IRB No. 2312-134-1496), and the requirement for informed consent was waived owing to the retrospective design of the study. The study was conducted in compliance with the 1964 Declaration of Helsinki.

### 2.2. Definitions

Baseline anthropometric data, including age, height (m), and weight (kg), were collected on the day of screening. Data on health behaviors were also collected on the day of screening through a self-reported questionnaire inquiring about smoking status (never, former, or current), alcohol consumption (none, <30 g/day, or ≥30 g/day), and regular exercise (high-intensity activity ≥3 times/week or moderate-intensity activity ≥5 times/week). The history of HRT (no history, <2 years, between 2 and 5 years, or 5 years or longer) was collected using a self-administered questionnaire at the time of national healthcare screening, along with information on other female reproductive factors such as age at menarche, age at menopause, parity (none, 1, 2, or more), breastfeeding history (no history, <6 months, between 6 and 12 months, or 1 year or longer), and history of OC use (no history, <1 year, or 1 year or longer).

Comorbidities, including diabetes mellitus, dyslipidemia, hypertension, chronic kidney disease, and autoimmune diseases, were identified using the International Classification of Diseases, 10th revision codes in the health claims data or results from the healthcare screening examination. The definitions of each comorbidity are summarized in Table S2, Supplemental Digital Content, https://links.lww.com/MD/R454. The primary outcome was a diagnosis of achalasia, which was identified using the International Classification of Diseases, 10th revision code K22.0.

### 2.3. Statistical analysis

Continuous variables were represented as mean ± SD and compared using analysis of variance, while categorical variables were represented as proportions and compared using the chi-square test. The covariates were categorized as shown in Table 1. The incidence rate (IR) of achalasia was calculated by dividing the number of cases by 1000 person-years at risk. The risk of achalasia was calculated based on the history of HRT (with or without a history of HRT) and according to the duration of HRT received (no, <2 years, between 2 and 5 years, or 5 years or longer) using 5 different multivariable Cox proportional hazard models. Model 1 was a crude nonadjusted model, whereas model 2 was adjusted for age. Model 3 was adjusted for age, body mass index, income, and lifestyle risk factors (alcohol consumption, smoking status, and regular exercise). Model 4 was additionally adjusted for comorbidities, such as diabetes mellitus, hypertension, dyslipidemia, chronic kidney disease, and autoimmune diseases. Model 5 was adjusted for female reproductive factors related to sex hormone exposure (age at menarche, age at menopause, history of OC use, number of live births, and breastfeeding history), as well as factors included in model 4. The results were presented as adjusted hazard ratios (aHRs) and 95% confidence intervals (CIs). The unadjusted cumulative incidence of achalasia stratified by the history and duration of HRT was plotted using Kaplan–Meier curves and compared using the log-rank test. Sensitivity analysis was performed by calculating the aHRs when the study period ended on December 31, 2014. Subgroup analyses were done according to age (<65 vs ≥65 years), income (lowest quadrant [Q1] + medical aid vs higher 3 quadrants [Q2–4]), history of autoimmune disease, and female reproductive factors.

**Table 1:** Baseline characteristics of the study population.

	No history of HRT (N = 1,101,080)	HRT < 2 yr (N = 121,740)	HRT 2 to <5 yr (N = 49,846)	HRT ≥ 5 yr (N = 37,980)		*P* value
Age (yr)	62.21 ± 8.64	58.23 ± 6.69	58.73 ± 6.31		60.68 ± 5.95	<.0001
BMI (kg/m^2^)	24.25 ± 3.2	23.91 ± 2.91	23.69 ± 2.8		23.82 ± 2.75	<.0001
Comorbidities						
Autoimmune disease (%)*	5571 (0.51)	846 (0.69)	374 (0.75)		312 (0.82)	<.0001
Diabetes mellitus (%)†	149,578 (13.58)	11,891 (9.77)	4479 (8.99)		3710 (9.77)	<.0001
Hypertension (%)	521,324 (47.35)	46,168 (37.92)	19,396 (38.91)		16,258 (42.81)	<.0001
Dyslipidemia (%)	362,913 (32.96)	39,830 (32.72)	15,389 (30.87)		11,387 (29.98)	<.0001
Chronic kidney disease (%)	136,478 (12.39)	11,110 (9.13)	4887 (9.8)		4754 (12.52)	<.0001
Cigarette smoking						<.0001
Never (%)	1,062,360 (96.48)	116,370 (95.59)	47,554 (95.4)		36,254 (95.46)	
Ex-smoker (%)	10,553 (0.96)	1799 (1.48)	794 (1.59)		659 (1.74)	
Current smoker (%)	28,167 (2.56)	3571 (2.93)	1498 (3.01)		1067 (2.81)	
Alcohol consumption						<.0001
None (%)	975,757 (88.62)	101,976 (83.77)	41,459 (83.17)		32,086 (84.48)	
Mild (<30 g/d) (%)	120,270 (10.92)	18,971 (15.58)	8045 (16.14)		5642 (14.86)	
Heavy (≥30 g/d) (%)	5053 (0.46)	793 (0.65)	342 (0.69)		252 (0.66)	
Regular exercise (%)	191,270 (17.37)	27,314 (22.44)	12,224 (24.52)		9962 (26.23)	<.0001
Income, lowest Q1	235,727 (21.41)	26,730 (21.96)	10,545 (21.16)		7617 (20.06)	<.0001
Age at menarche (yr)	16.51 ± 1.84	16.2 ± 1.85	16.17 ± 1.83		16.28 ± 1.84	<.0001
Age at menopause (yr)	49.98 ± 3.95	50 ± 3.91	49.87 ± 4.09		49.28 ± 4.67	<.0001
Total reproductive span (yr)	33.48 ± 4.38	33.8 ± 4.2	33.7 ± 4.33		32.99 ± 4.87	<.0001
Number of live births						<.0001
None (%)	16,618 (1.51)	2831 (2.33)	1304 (2.62)		1121 (2.95)	
1 (%)	59,859 (5.44)	10,201 (8.38)	4349 (8.72)		3144 (8.28)	
≥2 (%)	1,024,603 (93.05)	108,708 (89.3)	44,193 (88.66)		33,715 (88.77)	
Breastfeeding history (%)						<.0001
None	64,789 (5.88)	10,627 (8.73)	4785 (9.6)		3672 (9.67)	
<6 mo	64,225 (5.83)	10,424 (8.56)	4309 (8.64)		2901 (7.64)	
<12 mo	180,648 (16.41)	24,281 (19.94)	10,189 (20.44)		6573 (17.31)	
≥1 yr	791,418 (71.88)	76,408 (62.76)	30,563 (61.31)		24,834 (65.39)	
Oral contraceptives history (%)						<.0001
None	951,345 (86.4)	91,173 (74.89)	37,334 (74.9)		28,124 (74.05)	
<1 yr	91,300 (8.29)	19,330 (15.88)	6388 (12.82)		4715 (12.41)	
≥1 yr	58,435 (5.31)	11,237 (9.23)	6124 (12.29)		5141 (13.54)	
Follow-up duration (yr)	11.83 ± 2.08	12.19 ± 1.32	12.19 ± 1.33		12.17 ± 1.39	<.0001

Continuous variables (mean ± standard deviation); categorical variables: numbers (percentage).

BMI = body mass index, HRT = hormone replacement therapy.

* Diagnosed with any of the following autoimmune diseases, defined in Table S1: rheumatoid arthritis, ankylosing spondylitis, systemic lupus erythematosus, Sjögren syndrome, Behçet disease, dermatopolymyositis, systemic sclerosis, and other overlap syndrome.

† Type II diabetes mellitus.

All statistical analyses were performed using SAS version 9.4 (SAS Institute, Cary). Statistical significance was set at a 2-sided *P* value < .05.

## 3. Results

### 3.1. Baseline characteristics

The baseline characteristics of the study population according to the duration of HRT received (no, <2 years, between 2 and 5 years, or 5 years or longer) are summarized in Table 1. Of the 1,310,646 participants analyzed, 1,101,080 (84.01%) reported never having received HRT. Among the subjects who received HRT, 121,740 (9.29%) reported receiving HRT for <2 years, 49,846 (3.80%) for between 2 and 5 years, and 37,980 (2.90%) for 5 years or longer. Compared with subjects with no history of HRT, those who received HRT showed a higher prevalence of autoimmune diseases but a lower prevalence of diabetes mellitus, hypertension, and dyslipidemia. Individuals without a history of HRT tended to be nonsmokers and nondrinkers. Regular exercise demonstrated a linear association with HRT duration. Those without a history of HRT were more likely to have breastfed for over 1 year, be multiparous, and have no history of OC use. Age at menopause and age at menarche did not show a consistent trend.

### 3.2. Association with HRT and risk of achalasia

**Figure 2.**
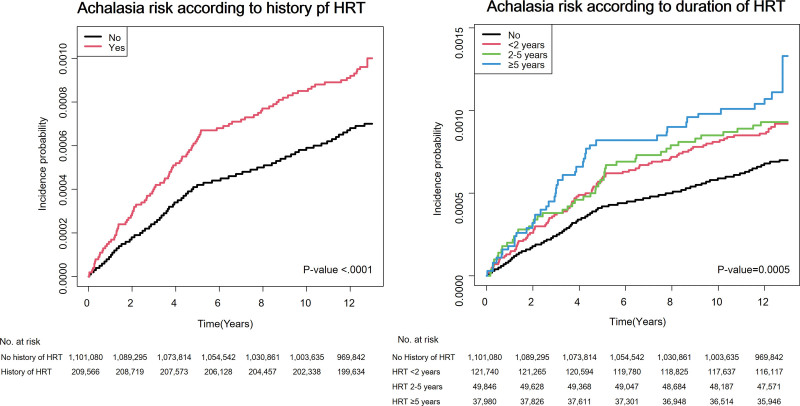
Kaplan–Meier curve of the cumulative incidence of achalasia according to the history and duration of HRT. HRT = hormone replacement therapy.

The aHRs and 95% CIs of achalasia according to HRT history are presented in Table 2. Overall, the IR of achalasia in individuals who had never received HRT was 0.056 per 1000 person-years, with a cumulative incidence of 731 of 1,101,080 subjects. By contrast, the IR in the group with a history of HRT was 0.077 per 1000 person-years, with a cumulative incidence of 196 of 209,566 subjects. In all 5 models, the risk of developing achalasia was significantly higher in those who received HRT (aHR in model 5: 1.47; 95% CI: 1.25–1.73). The same trend is shown in the Kaplan–Meier curve in Figure 2 (*P* value < .0001).

**Table 2:** Risk of achalasia according to duration of hormone replacement therapy.

Group	IR*	HR (95% CI)				
		Model 1	Model 2	Model 3	Model 4	Model 5
No history of HRT	0.056	1 (Ref.)	1 (Ref.)	1 (Ref.)	1 (Ref.)	1 (Ref.)
History of HRT	0.077	1.38 (1.18–1.61)	1.48 (1.26–1.73)	1.48 (1.26–1.74)	1.47 (1.25–1.73)	1.47 (1.25–1.73)
*P* value		<.0001	<.0001	<.0001	<.0001	<.0001
No history of HRT	0.056	1 (Ref.)	1 (Ref.)	1 (Ref.)	1 (Ref.)	1 (Ref.)
HRT < 2 yr	0.073	1.31 (1.07–1.60)	1.41 (1.15–1.74)	1.42 (1.15–1.74)	1.41 (1.15–1.73)	1.41 (1.14–1.73)
HRT 2 to <5 yr	0.076	1.36 (1.01–1.83)	1.46 (1.08–1.97)	1.46 (1.08–1.97)	1.45 (1.08–1.96)	1.46 (1.08–1.97)
HRT ≥ 5 yr	0.091	1.69 (1.19–2.22)	1.68 (1.23–2.30)	1.69 (1.24–2.31)	1.69 (1.24–2.31)	1.69 (1.23–2.31)
*P* value		<.0001	<.0001	<.0001	<.0001	<.0001

Model 1: not adjusted.

Model 2: adjusted for age.

Model 3: model 2 + adjusted for BMI, income, smoking status, drinking status, and regular exercise.

Model 4: model 3 + adjusted for diabetes mellitus, hypertension, dyslipidemia, chronic kidney disease, and autoimmune disease.

Model 5: model 4 + adjusted for age at menarche, age at menopause, number of live births, breastfeeding history, and oral contraceptives history.

CI = confidence interval, HR = hazard ratio, HRT = hormone replacement therapy, IR = incidence rate.

* Number of cases per 1000 person-years.

When analyzed according to HRT duration, the risk of developing achalasia increased in a dose-dependent manner. The IR of achalasia was 0.073, 0.076, and 0.091 per 1000 person-years for HRT durations of <2 years, between 2 and 5 years, and 5 years or longer, respectively. The aHRs were 1.41 (95% CI: 1.14–1.73) for those who received HRT for <2 years, 1.46 (95% CI: 1.08–1.97) for between 2 and 5 years, and 1.69 (95% CI: 1.23–2.31) for 5 years or longer. The cumulative incidence of achalasia also increased proportionally with HRT duration in Figure 2 (*P* value = .0005).

To validate these results, a sensitivity analysis was conducted by calculating aHRs with a shorter follow-up duration ending on December 31, 2014, as detailed in Table S3, Supplemental Digital Content, https://links.lww.com/MD/R454. In this analysis, a history of HRT was associated with a higher risk of achalasia, with an aHR of 1.60 (95% CI: 1.34–1.96), which is consistent with the results presented in Table 2. Moreover, aHRs were highest in those who received HRT for longer than 5 years, which resembled the trends observed in Table 2 and Figure 2.

To account for the chronic and slowly progressing nature of achalasia, an additional sensitivity analysis was performed by extending the lag period to 3 years. Specifically, individuals who were diagnosed with achalasia or died within 3 years following their general health screening were excluded. aHRs were then calculated according to both HRT history and duration (Table S4, Supplemental Digital Content, https://links.lww.com/MD/R454). The association was modestly attenuated but remained generally consistent with the primary analysis.

### 3.3. Subgroup analyses

The association between a history of HRT and the risk of developing achalasia differentiated by subgroups such as age, income, female reproductive factors, and a history of autoimmune disease is shown in Table 3. Although low socioeconomic status is known to be associated with a higher risk of achalasia,^[13]^ the interaction between income and HRT was not significant in our study, with a *P* value of .85. Similarly, female reproductive factors that may influence endogenous and exogenous hormone exposure, such as age at menarche and menopause, use of OCs, parity, and breastfeeding history, showed no significant interaction with a history of HRT in affecting the risk of achalasia. Notably, while aHR in the HRT group was higher (aHR: 2.73; 95% CI: 0.61–12.20) among those with underlying autoimmune disease, compared to those without such diseases (aHR: 1.46; 95% CI: 1.24–1.72), the *P* for interaction was not significant. Overall, the risk of developing achalasia was consistently higher in participants with a history of HRT, regardless of age, income, female reproductive factors, or a history of autoimmune disease.

**Table 3:** Subgroup analysis according to age, income, and female reproductive factors.

	History of HRT	IR*	Adjusted HR† (95% CI)	*P* for interaction
Age (yr)				
<65	No	0.050	1 (ref.)	.422
	Yes	0.071	1.42 (1.17–1.71)	
≥65	No	0.067	1 (ref.)	
	Yes	0.103	1.64 (1.20–2.24)	
Income‡				
Q2–4	No	0.0565	1 (Ref.)	.853
	Yes	0.0777	1.48 (1.24–1.78)	
Q1 + medical aid	No	0.0548	1 (Ref.)	
	Yes	0.0731	1.43 (1.01–2.03)	
Age at menarche (yr)				
≤12	No	0.0419	1 (Ref.)	.943
	Yes	0.0571	1.39 (0.27–7.14)	
>12	No	0.0562	1 (Ref.)	
	Yes	0.0770	1.47 (1.25–1.73)	
Age at menopause (yr)				
<50	No	0.059	1 (Ref.)	.323
	Yes	0.070	1.32 (1.01–1.73)	
≥50	No	0.055	1 (Ref.)	
	Yes	0.081	1.56 (1.28–1.91)	
Oral contraceptives history (%)				
No or <1 yr	No	0.056	1 (Ref.)	.0515
	Yes	0.081	1.54 (1.30–1.82)	
≥1 yr	No	0.057	1 (Ref.)	
	Yes	0.044	0.80 (0.42–1.52)	
Number of live births				
None	No	0.071	1 (Ref.)	.636
	Yes	0.078	1.15 (0.42–3.20)	
≥1	No	0.056	1 (Ref.)	
	Yes	0.077	1.48 (1.26–1.75)	
Breastfeeding history				
No	No	0.060	1 (Ref.)	.517
	Yes	0.073	1.24 (0.71–2.15)	
Yes	No	0.056	1 (Ref.)	
	Yes	0.077	1.50 (1.26–1.77)	
History of autoimmune disease§				
No	No	0.056	1 (Ref.)	.415
	Yes	0.076	1.46 (1.24–1.72)	
Yes	No	0.063	1 (Ref.)	
	Yes	0.166	2.73 (0.61–12.20)	

BMI = body mass index, CI = confidence interval, HR = hazard ratio, HRT = hormone replacement therapy, IR = incidence rate.

* Number of cases per 1000 person-years.

† Adjusted for age, BMI, income, smoking status, drinking status, regular exercise, diabetes mellitus, hypertension, dyslipidemia, chronic kidney disease, autoimmune disease, age at menarche, age at menopause, number of live births, breastfeeding history, and oral contraceptives history.

‡ Income grade was divided into 4 quadrants, with Q1: lowest and Q4: highest.

§ Diagnosed with any of the following autoimmune diseases, defined in Table S1: rheumatoid arthritis, ankylosing spondylitis, systemic lupus erythematosus, Sjögren syndrome, Behçet disease, dermatopolymyositis, systemic sclerosis, and other overlap syndrome.

## 4. Discussion

Achalasia is a rare but debilitating disease, affecting 7 to 32 per 100,000 individuals worldwide.^[5]^ Although most studies show largely equal prevalence in both sexes, several epidemiologic studies have shown a slight female predominance.^[17,18]^ This suggests that female sex hormones contribute to the development of this condition. However, to the best of our knowledge, no other studies have explored the association between exposure to female sex hormones and the development of achalasia. Our study presents compelling evidence for the association between a history of HRT and the risk of developing achalasia in postmenopausal women. Our findings indicate that the risk of achalasia increases in a dose-dependent manner with the duration of HRT, as shown by the increase in aHRs with longer HRT exposure. This association remained constant in subgroups divided according to potential confounding factors, including age, income, female reproductive factors, and history of autoimmune diseases, suggesting that a history of HRT is an independent risk factor for the development of achalasia in postmenopausal women.

The exact mechanism through which HRT may increase the risk of achalasia likely involves hormonal effects on gastrointestinal motility. Previous studies have shown sex differences in gastrointestinal motility, with females more prone to disturbances such as gastroparesis and constipation.^[19–21]^ Estrogen and progesterone have been shown to decrease gastric smooth muscle contractility via the nitric oxide–cyclic guanosine monophosphate pathway, which may contribute to the higher prevalence of gastroparesis in females.^[22,23]^ However, studies examining the effects of female sex hormones on esophageal motility are limited. One animal study demonstrated that estradiol reduces esophageal muscle contraction by regulating calcium-related proteins, which appears to contradict our findings.^[24]^ While the effect of estrogen on esophageal motility in humans remains unclear, this study suggests a possible molecular mechanism through which estrogen may influence esophageal function. Further experimental research is needed to elucidate the exact role of estrogen in esophageal motility disorders.

Combined with their direct effects on esophageal motility, the immune-modulating properties of female sex hormones may significantly contribute to the increased risk of achalasia. Recent studies suggest that the pathogenesis of achalasia possibly involves autoimmune reactions triggered by latent viral infections in genetically predisposed individuals, which leads to the disruption of myenteric plexus ganglion cells in the lower esophagus.^[5,9,11,12]^ Studies have also shown that levels of various cytokines, such as interleukin-2, chemokines, and complement proteins, are elevated in patients with achalasia, contributing to a pro-inflammatory environment.^[25–27]^ Infiltration of T cells in the myenteric plexus of the lower esophageal sphincter in patients with achalasia has been repeatedly documented, suggesting a significant role for T cells in the pathogenesis of achalasia.^[10,28,29]^ Furthermore, eosinophil and mast cell infiltration has been widely observed in the esophageal myenteric plexus of achalasia patients.^[28–30]^ On the other hand, the effect of female sex hormones on the immune system is complex, having both pro-inflammatory and anti-inflammatory effects depending on the immunologic context.^[16,31]^ Estrogen has been shown to activate T-cell-mediated autoimmune responses in animal models of colitis and autoimmune thyroiditis.^[32,33]^ While progesterone generally exhibits anti-inflammatory properties, it has also been shown to stimulate eosinophilic immune reactions.^[34,35]^ Taken together, these findings suggest that exposure to exogenous female sex hormones may exacerbate autoimmune responses mediated by T cells and eosinophils against the esophageal myenteric plexus, thereby increasing the risk of achalasia.

Additionally, alterations in gastrointestinal barrier function may contribute to the increased risk of achalasia observed in HRT users. HRT and OC have previously been linked to an increased risk of inflammatory bowel disease, and one proposed mechanism involves estrogen-induced disruption of mucosal barrier integrity.^[36–38]^ Extending this hypothesis to achalasia, a recent study analyzing esophageal biopsy samples from patients with achalasia revealed changes in key signaling pathways within the esophageal epithelium.^[39]^ These findings suggest that exogenous sex hormones, such as those used in HRT, may impair epithelial barrier function and thereby contribute to disease development.

A higher prevalence of autoimmune conditions has been observed in patients with achalasia compared with the general population.^[40,41]^ Conversely, previous studies have also reported an increased risk of achalasia in patients with multiple sclerosis, an autoimmune disease affecting the central nervous system.^[42]^ Previous studies have also shown that HRT influences the development and activity of various autoimmune diseases. For instance, HRT increases the risk of systemic lupus erythematosus and can induce mild-to-moderate flares,^[43]^ while it shows a protective effect in rheumatoid arthritis.^[44]^ Our data also showed that subjects who received HRT demonstrated a higher prevalence of autoimmune diseases than those who had never received HRT. The association of HRT with autoimmunity, along with the link between autoimmunity and achalasia, further supports our hypothesis that HRT may increase the risk of achalasia through autoimmune mechanisms.

There are several limitations in our study. One limitation of our study is that the diagnostic methods were not fully standardized, since the identification of achalasia was based on the claims data. However, in a previous Korean population-based study, the validity of achalasia cases identified by the K22.0 code was confirmed through review of hospital records and manometry findings.^[6]^ In our own institutional review, 244 patients were assigned the K22.0 code between 2017 and 2025, and 35 of them had already been diagnosed at outside hospitals. Among the remaining 209 patients, 106 (50.7%) were diagnosed based on compatible manometry findings, and 70 (33.5%) were diagnosed based on typical endoscopic or radiologic features (upper endoscopy or upper gastrointestinal series) with exclusion of structural disease. The remaining 33 patients (15.8%) did not meet diagnostic criteria or had only clinical suspicion without confirmatory findings. Thus, 176 of 209 patients (84.2%) had a confirmed diagnosis of achalasia. Furthermore, among 75 patients treated with pneumatic dilation or POEM during the same period, 98.7% (74/75) had also received the K22.0 code. These findings collectively support the reasonable validity of using this diagnostic code to identify achalasia cases in claims-based research. Second, the lack of detailed information on the formulation, dose, and age at HRT use is a limitation of our study. However, considering that only those who had reached natural menopause were included, since women who had undergone hysterectomy were excluded, most would have received an estrogen-plus-progesterone regimen. Nevertheless, considering the different immunologic effects of estrogen and progesterone and their dependence on hormone concentrations, a more detailed analysis of the formulation and dosing of HRT could have provided deeper insights into the mechanism underlying the increased risk of achalasia in HRT users. Thus, further clinical and preclinical studies comparing the effects of estrogen and progesterone on achalasia development are warranted. The third limitation is that the history of HRT was acquired through self-administered questionnaires, which introduced the possibility of recall bias. However, it has been reported that compliance with HRT in the Korean population is as low as 61% after 1 year of treatment and 50% after 3 years.^[45]^ This suggests that self-reported information on HRT may be more accurate than prescription data. Another limitation of this study is the lack of information on medications known to affect esophageal motility, such as opioids, calcium channel blockers, anticholinergics, and certain antidepressants. These agents can induce secondary esophageal dysmotility and may act as potential confounders. However, drug-induced motility disturbances are typically reversible upon discontinuation, whereas achalasia is a chronic, progressive disorder with distinct clinical, radiological, and manometric features. In clinical practice, achalasia can usually be differentiated from transient drug effects through careful patient history, imaging studies, and high-resolution manometry. Finally, although our study found an association between HRT use and achalasia risk, this does not imply causality. Due to the chronic and insidious nature of achalasia, it is often difficult to be diagnosed in the early stage. To address these issues, we implemented a 1-year lag period in the primary analysis and conducted an additional sensitivity analysis using a 3-year lag period, which showed consistent results.

Nevertheless, the strength of our study lies in its large sample size, which represents all postmenopausal women in South Korea, and its longitudinal, nationwide, population-based cohort design. Moreover, the robust collection of anthropometric data, comorbidities, including autoimmune diseases, and lifestyle factors allowed us to adjust for possible confounding factors, thereby strengthening the results of our study. Using this powerful database, we were able to show a strong, dose-dependent association between HRT and achalasia.

In conclusion, we observed a significant dose-dependent increase in the risk of achalasia associated with prolonged HRT in this extensive nationwide cohort study. This association remained consistent across subgroups defined by age, income, female reproductive factors, and a history of autoimmune disease. To our knowledge, this is the first study to suggest HRT as an independent modifiable risk factor for the development of achalasia. This finding is of epidemiological importance and highlights the need for further research into the role of sex-related factors in the pathogenesis of achalasia. Clinicians should remain vigilant to symptoms suggestive of esophageal dysmotility in women with a prolonged history of HRT.

## Author contributions

**Conceptualization:** Mi Jin Oh, Yoon Jin Choi, Kyungdo Han, Soo-Jeong Cho.

**Funding acquisition:** Soo-Jeong Cho.

**Methodology:** Kyungdo Han, Kyu-Na Lee.

**Visualization:** Mi Jin Oh.

**Supervision:** Yoon Jin Choi, Kyungdo Han, Hyunsoo Chung, Sang Gyun Kim, Soo-Jeong Cho.

**Data curation:** Kyungdo Han.

**Formal analysis:** Kyu-Na Lee.

**Software:** Kyu-Na Lee.

**Writing – original draft:** Mi Jin Oh.

**Writing – review & editing:** Mi Jin Oh, Yoon Jin Choi, Gi Hong Park, Bokyung Kim, Soo-Jeong Cho.

## Supplementary Material


