# Supplementary material for: Hormone replacement therapy and the risk of achalasia in postmenopausal women: A nationwide cohort study
Source: Medicine (Baltimore). 2026 Feb 28;105(9):e47834. doi: 10.1097/MD.0000000000047834 (PMC12956221; doi:10.1097/MD.0000000000047834)
Supplement: Supplementary file 1 [file medi-105-e47834-s001.docx]

**Supplemental Digital Content 1**. Baseline characteristics of patients excluded due to missing data, compared to enrollees

|  | Enrollees  (N=1,314,676) | Excluded due to missing data  (N=275,393) |
| --- | --- | --- |
| Age (years) | 61.7±8.45 | 62.59±8.84 |
| BMI (kg/m^2^) | 24.18±3.16 | 24.25±3.2 |
| Comorbidities |  |  |
| Autoimmune disease  (%)^a^ | 8,308 (0.63) | 1,696 (0.62) |
| Diabetes mellitus (%)^b^ | 171,182 (13.02) | 38,244 (13.91) |
| Hypertension (%) | 603,751 (45.92) | 132,218 (48.13) |
| Dyslipidemia (%) | 429,898 (32.7) | 88,919 (32.7) |
| Chronic kidney disease  (%) | 157,705 (12) | 37,127 (13.51) |
| Cigarrette smoking |  |  |
| Never (%) | 1,266,428 (96.33) | 254,099 (95.27) |
| Ex-smoker (%) | 13,804 (1.05) | 4,480 (1.68) |
| Current smoker (%) | 34,444 (2.62) | 8,134 (3.05) |
| Alcohol consumption |  |  |
| None (%) | 1,154,813 (87.84) | 223,835 (87.41) |
| Mild (<30g/day) (%) | 153,422 (11.67) | 30,831 (12.04) |
| Heavy (≥30g/day) (%) | 6,441 (0.49) | 1,408 (0.55) |
| Regular exercise (%) | 241,506 (18.37) | 43,109 (16.9) |
| Income, Lowest Q1 | 285,153 (21.69) | 55,069 (21.99) |
| Age at menarche (years) | 16.46±1.84 | 16.52±1.82 |
| Age at menopause (years) | 49.96±3.98 | 49.84±4.01 |
| Total reproductive span (years) | 33.5±4.38 | 33.31±4.41 |
| Number of live births |  |  |
| None (%) | 21955 (1.67) | 19,786 (7.27) |
| 1 (%) | 77697 (5.91) | 16,492 (6.06) |
| ≥2 (%) | 1215024 (92.42) | 235,750 (86.66) |
| Breastfeeding history  (%) |  |  |
| None | 84007 (6.39) | 17,903 (7.03) |
| <6 months | 82035 (6.24) | 17,020 (6.68) |
| <12 months | 222444 (16.92) | 46,318 (18.18) |
| ≥1 year | 926190 (70.45) | 173,543 (68.11) |
| Oral contraceptives  history (%) |  |  |
| None | 1111558 (84.55) | 154,760 (81.62) |
| <1 year | 122001 (9.28) | 19,682 (10.38) |
| ≥1 year | 81115 (6.17) | 15,167 (8) |
| History of HRT |  |  |
| None | 1104855 (84.04) | 160,251 (83.83) |
| <2 years | 121870 (9.27) | 17,491 (9.15) |
| 2 to <5 years | 49957 (3.8) | 7,455 (3.9) |
| ≥5 years | 37994 (2.89) | 5,964 (3.12) |

**Supplemental Digital Content 2.** Operational definitions of comorbidities

| **Autoimmune disease** |  |  |
| --- | --- | --- |
| Rheumatoid arthritis | M05 and RID code (V223) | Admission or outpatient department≥1 |
| Ankylosing spondylitis | M45 and RID code (V140) | Admission or outpatient department≥1 |
| Systemic lupus erythematosus | M32 and RID code (V136) | Admission or outpatient department≥1 |
| Sjogren’s syndrome | M35.0 and RID code (V139) | Admission or outpatient department≥1 |
| Behcet’s disease | M35.2 and RID code (V139) | Admission or outpatient department≥1 |
| Dermatopolymyositis | M33, M33.1, M33.2, M33.9 and RID code (V137) | Admission or outpatient department≥1 |
| Systemic sclerosis | M34 and RID code (V138) | Admission or outpatient department≥1 |
| Other overlap syndrome | M35.1 and RID code (V139) | Admission or outpatient department≥1 |
| History of autoimmune disease | Diagnosis of any autoimmune disease stated above by the following definitions | |
| **Other comorbidities** | **ICD-10 codes and definition** | **Diagnostic definition** |
| Diabetes mellitus | E11-E14; and minimum one prescription of anti-diabetic drugs (sulfonylurea, metformin, meglitinide, thiazolidinedione, dipeptidyl peptidase-4 inhibitor, α-glucosidase inhibitor, and insulin). | Admission≥1 or outpatient department≥1 |
|  | Or fasting glucose level ≥ 126 mg/dL | Index health examination |
| Hypertension | I10-I13, I15; and minimum one prescription of anti-hypertensive medication (thiazide, loop diuretic, aldosterone antagonist, alpha-/beta-blocker, calcium channel blocker, angiotensin-converting enzyme inhibitor, and angiotensin II receptor blocker). | Admission≥1 or outpatient department≥2 |
|  | or systolic/diastolic blood pressure ≥ 140/90 mmHg | Index health examination |
| Dyslipidemia | E78 | Admission or outpatient department≥1 |
|  | Or Total cholesterol ≥ 240 mg/dL | Index health examination |
| Chronic kidney disease | Estimated glomerular filtration rate <60 ml/min/1.73m^2^ | Index health examination |

**Supplemental Digital Content 3.** Sensitivity analysis: Risk of achalasia according to history and duration of hormone replacement therapy with a restricted 5-year follow-up period

| Group | IR^a^ | HR (95% CI) | | | | |
| --- | --- | --- | --- | --- | --- | --- |
|  |  | Model 1 | Model 2 | Model 3 | Model 4 | Model 5 |
| No history of HRT | 0.0892 | 1 (Ref.) | 1 (Ref.) | 1 (Ref.) | 1 (Ref.) | 1 (Ref.) |
| History of HRT | 0.1330 | 1.49  (1.24-1.79) | 1.60  (1.33-1.94) | 1.61  (1.33-1.94) | 1.60  (1.32-1.93) | 1.62  (1.34-1.96) |
| P value |  | <.0001 | <.0001 | <.0001 | <.0001 | <.0001 |
| No history of HRT | 0.0892 | 1 (Ref.) | 1 (Ref.) | 1 (Ref.) | 1 (Ref.) | 1 (Ref.) |
| HRT < 2 years | 0.1198 | 1.34  (1.05-1.71) | 1.46  (1.14-1.86) | 1.46  (1.14-1.87) | 1.44  (1.13-1.85) | 1.46  (1.14-1.87) |
| HRT 2 to < 5 years | 0.1241 | 1.39  (0.97-1.99) | 1.50  (1.04-2.14) | 1.50  (1.05-2.15) | 1.49  (1.04-2.14) | 1.52  (1.06-2.18) |
| HRT ≥ 5 years | 0.1863 | 2.09  (1.49-2.91) | 2.16  (1.55-3.02) | 2.17  (1.55-3.04) | 2.19  (1.56-3.06) | 2.24  (1.60-3.13) |
| P-value |  | <.0001 | <.0001 | <.0001 | <.0001 | <.0001 |

**Supplemental Digital Content 4**. Sensitivity analysis: Risk of achalasia according to history and duration of hormone replacement therapy with a 3-year lag period

| Group | IR^a^ | HR (95% CI) | | | | |
| --- | --- | --- | --- | --- | --- | --- |
|  |  | Model 1 | Model 2 | Model 3 | Model 4 | Model 5 |
| No history of HRT | 0.050 | 1 (Ref.) | 1 (Ref.) | 1 (Ref.) | 1 (Ref.) | 1 (Ref.) |
| History of HRT | 0.064 | 1.29  (1.07-1.56) | 1.38  (1.14-1.67) | 1.38 (1.14- 1.67) | 1.38  (1.14-1.67) | 1.37  (1.13- 1.67) |
| P value |  | 0.0075 | 0.001 | 0.001 | 0.001 | 0.0013 |
| No history of HRT | 0.050 | 1 (Ref.) | 1 (Ref.) | 1 (Ref.) | 1 (Ref.) | 1 (Ref.) |
| HRT < 2 years | 0.061 | 1.24  (0.98-1.58) | 1.34  (1.05-1.71) | 1.34  (1.05-1.71) | 1.34  (1.05-1.71) | 1.33  (1.04-1.40) |
| HRT 2 to < 5 years | 0.059 | 1.20  (0.83-1.73) | 1.28  (0.89-1.85) | 1.28  (0.88-1.85) | 1.28  (0.88-1.85) | 1.28  (0.88-1.85) |
| HRT ≥ 5 years | 0.078 | 1.58  (1.09-2.28) | 1.62  (1.12-2.34) | 1.63  (1.13- 2.35) | 1.63  (1.13-2.36) | 1.62  (1.12-2.35) |
| P-value |  | 0.0307 | 0.0069 | 0.0068 | 0.0066 | 0.0088 |
